# A phase 1 study of durvalumab as monotherapy or combined with tremelimumab with or without azacitidine in patients with myelodysplastic syndrome

**DOI:** 10.1007/s00277-024-06081-4

**Published:** 2025-03-28

**Authors:** Guillermo Garcia-Manero, Manila Gaddh, Uwe Platzbecker, R. Coleman Lindsley, Sarah M. Larson, Timothy Chevassut, Pierre Fenaux, Rami Komrokji, Roger Lyons, Aref Al-Kali, Yu Jiang, John Bothos, Danielle M. Townsley, Amer M. Zeidan

**Affiliations:** 1 MD Anderson Cancer Center, Houston, TX USA; 2 Department of Hematology and Medical Oncology, Emory University School of Medicine, Atlanta, GA USA; 3 University Hospital Leipzig, Leipzig, Germany; 4 Dana-Farber Cancer Institute, Boston, MA USA; 5 David Geffen School of Medicine at UCLA, Los Angeles, CA USA; 6 Brighton and Sussex Medical School, University of Sussex, Brighton, UK; 7 Hôpital St Louis/Université Paris, Paris, France; 8 H Lee Moffitt Cancer Center, Tampa, FL USA; 9 Texas Oncology and US Oncology Research, San Antonio, TX USA; 10 Mayo Clinic, Rochester, MN USA; 11 Clinical Pharmacology and Safety Sciences, BioPharmaceuticals Research and Development, AstraZeneca, Gaithersburg, MD USA; 12 Oncology Research and Development, AstraZeneca, Gaithersburg, MD USA; 13 Section of Hematology, Department of Internal Medicine, Yale School of Medicine and Yale Comprehensive Cancer Center, New Haven, CT USA

**Keywords:** Acute myeloid leukemia, CTLA-4, Durvalumab, First-in-human, MDS, PD-L1

## Abstract

Upregulation of programmed death ligand-1 (PD-L1) has been observed in patients with MDS, and its expression on myeloblasts is associated with progression to AML. This open-label, phase 1 study evaluated the safety and tolerability of the PD-L1 antibody durvalumab as monotherapy (part 1) and in combination with tremelimumab, with or without azacitidine (part 2), in patients with MDS who progressed following hypomethylating agent treatment. Sixty-seven adults with MDS were enrolled (part 1, 40 with low/intermediate-1 or intermediate-2/high IPSS risk status; part 2, 27 with intermediate-2/high IPSS risk status). Primary safety endpoints included dose-limiting toxicities (DLTs) and treatment-emergent adverse events (TEAEs). Secondary endpoints included evaluation of clinical outcomes, survival, and pharmacokinetics. Dose-limiting toxicities were experienced by no patients in part 1 and 3 patients (11%) in part 2. The most common treatment-emergent adverse events were diarrhea and fatigue (40% each) in part 1 and fatigue (44%) and anemia (37%) in part 2. In parts 1 and 2, 15% of patients experienced marrow complete response as their best overall response, according to IWG criteria. Hematologic improvement was observed in 35% and 30% of patients respectively in part 1 and part 2. The study was terminated early due to limited efficacy.

**Supplementary Information:**

The online version contains supplementary material available at 10.1007/s00277-024-06081-4.

## Introduction

Myelodysplastic syndromes (MDSs) are a heterogeneous group of clonal hematopoietic stem cell disorders typically diagnosed in patients who are over 60 years old [1, 2]. MDS is characterized by impaired hematopoiesis due to morphologic dysplasia in hematopoietic stem cells, leading to peripheral cytopenia. Depending on the stage of disease at diagnosis, MDS is often associated with a high risk for progression to acute myeloid leukemia (AML) [2–4].

MDS develops due to accumulation of genetic mutations that affect various cellular pathways including stress response, DNA damage repair, transcription, RNA splicing, epigenetics, and cytokine signaling, leading to the development of one or more malignant clones [2]. Commonly mutated genes in MDS include *TET2* (DNA methylation), *ASXL1*, *EZH2* (histone modification), *NRAS*, *KRAS*, *JAK2* (signaling factors), *GATA2* (transcription factors), and *SF3B1* (splicing factor) [2]. Multiple factors contribute to the pathogenesis of MDS, such as the development of immune dysregulation associated with alterations in natural killer cells, T cells, FOXP3^+^ regulatory T cells, and myeloid-derived suppressor cells [4].

The prognosis for patients with MDS and the risk of transformation to AML are based on the revised International Prognostic Scoring System.

(IPSS-R) [5]. The IPSS-R model categorizes patients based on cytopenias, cytogenetics, and bone marrow blast percentage into risk groups of very low risk, low risk, intermediate risk, high risk, and very high risk. Median survival rates for patients in each group after initial diagnosis are 8.8, 5.3, 3.0, 1.6, and 0.8 years, respectively [5]. For patients with high-risk disease, median overall survival (OS) is 5.6 months (95% confidence interval [CI], 5–7.2), with 1- and 2-year survival rates of 28.9% and 15.3%, respectively [6].

The only treatment option with curative potential for MDS is allogeneic hematopoietic stem cell transplantation, which is unavailable to most patients [7]. Currently available therapies, such as iron chelators, growth factors, lenalidomide, and hypomethylating agents (HMAs), are noncurative, with the aim of slowing disease progression and improving cytopenias [7]. HMAs, such as 5-azacitidine and 5-aza-2’deoxycitidine, are standard therapies for patients with high-risk MDS; however, treatment improves survival only marginally, and resistance to HMAs is common [4]. For patients with MDS who do not respond to HMAs, additional treatment options are limited.

Programmed death-ligand 1 (PD-L1) is expressed on several cancer and immune cell types and binds to programmed death-1 (PD-1) and CD80, negative regulators of T-lymphocyte activation [8]. The binding of PD-L1 to these receptors suppresses T-cell migration, proliferation, and cytotoxic mediator secretion, thereby inhibiting tumor cell death [8]. Preclinical studies demonstrated that elevated PD-L1 expression significantly reduced the anti-leukemic effect of CD8 + cytotoxic lymphocytes (CTLs) in an AML mouse model. The addition of an anti-PD-L1 antibody enhanced antitumor CD8 + CTL responses by preventing CD8 + exhaustion, decreasing AML burden, and increasing survival [9]. PD-L1 expression is upregulated in patients with MDS, and PD-1 signaling may be involved in MDS pathogenesis and the mechanisms underlying HMA resistance [4, 10]. Furthermore, expression of PD-L1 on myeloblasts is associated with transformation of MDS to acute myeloid leukemia (AML) [11].

Durvalumab is an immunoglobulin G1 (IgG1) anti-PD-L1 monoclonal antibody that is approved by the U.S. Food and Drug Administration for the treatment of unresectable non–small-cell lung cancer and extensive-stage small-cell lung cancer [12]. Tremelimumab is a human IgG2 monoclonal antibody that blocks the activity of cytotoxic T-lymphocyte–associated protein 4 (CTLA-4) to enhance T-cell activation. Preclinical studies have demonstrated that combination immune checkpoint inhibitor therapy using durvalumab and tremelimumab results in higher antitumor activity compared with either treatment alone [13, 14]. 

Several clinical trials are evaluating the safety and efficacy of monotherapy and combination therapies with immune checkpoint inhibitors for patients with MDS. A phase 2 trial is assessing the efficacy and safety of azacitidine in combination with durvalumab versus azacitidine alone in patients with high-risk MDS or AML. While the combination was tolerable, no significant difference in efficacy between the treatment groups was observed [15]. A phase 1b trial of atezolizumab, with or without azacitidine, in HMA-failure and HMA-naive patients with MDS, was terminated early due to unfavorable safety and limited efficacy [16]. Combinations of nivolumab or ipilimumab with azacitidine [17] and ipilimumab monotherapy following HMA failure [18] demonstrated manageable safety profiles and clinical activity in patients with MDS. Additional trials evaluating the PD-1 inhibitor pembrolizumab in patients with MDS have demonstrated its safety and tolerability, and combination therapy with azacitidine may have antitumor activity in patients who previously progressed on HMA therapy [19, 20].

This multicenter, open-label, phase 1 study evaluated the safety, tolerability, efficacy, and pharmacokinetics of durvalumab in patients with MDS who had disease progression following treatment with HMAs. In part 1 of the study, durvalumab was administered as monotherapy in patients with low/intermediate-1 and intermediate-2/high-risk MDS; and in part 2, durvalumab was administered in combination with tremelimumab, with or without azacitidine, in patients with intermediate-2/high-risk MDS.

## Methods

Eligible patients were ≥ 18 years of age, with pathologically confirmed MDS (< 20% blasts in bone marrow and/or peripheral blood). In part 1 of the study, patients with an International Prognostic Scoring System (IPSS) MDS risk status [21] of low, intermediate-1, intermediate-2, or high risk were enrolled. In part 2, enrollment was restricted to an IPSS risk status of intermediate-2 or high risk. Patients had either relapsed after an initial response to the most recent prior therapy or were unable to tolerate at least 4 cycles of prior 5-azacitidine or decitabine.

The study was conducted in accordance with the ethical principles originating in the Declaration of Helsinki and is consistent with the International Conference on Harmonisation and Good Clinical Practice guidelines, and applicable regulatory requirements. All patients provided written informed consent prior to participation. This study is registered with ClinicalTrials.gov, NCT02117219.

This phase 1, open-label, multicenter study enrolled patients at 15 study centers in France, Germany, the United Kingdom, and the United States. Part 1 consisted of a durvalumab monotherapy dose-escalation phase followed by a dose-expansion phase of two cohorts (low/intermediate-1 or intermediate-2/high risk patients). The recommended dose of durvalumab for part 1 dose expansion and for part 2 was 10 mg/kg every 2 weeks (Q2W), equivalent to a fixed dose of 1500 mg Q4W. Patients who experienced progressive disease (PD) on durvalumab monotherapy could receive add-on azacitidine (75 mg/m^2^ on days 1–7 of each 28-day cycle).

In part 2, patients were enrolled in 2 arms. Arm 1 was divided into 2 cohorts; cohort 1 received durvalumab 1500 mg every 4 weeks (Q4W) in combination with tremelimumab 25 mg Q4W and cohort 2 received durvalumab 1500 mg Q4W in combination with tremelimumab 75 mg Q4W. In Arm 2, patients were administered durvalumab 1500 mg Q4W in combination with tremelimumab 75 mg Q4W and azacitidine (75 mg/m^2^/day on days 1–7 of each 28-day cycle). Disease assessments were performed at baseline, Week 8, Week 16, and then every 16 weeks until end of treatment.

The primary endpoint was safety. Secondary endpoints included the evaluation of clinical outcomes according to the International Working Group (IWG) 2006 MDS response criteria; [22] and responses needed to last for at least 4 weeks. Patients were evaluated for dose-limiting toxicities (DLTs) in part 1 and in part 2, arm 1, and in the safety run-in (i.e., enrollment of the first 6 patients) of part 2, arm 2. Adverse events (AEs) were graded according to National Cancer Institute Common Terminology Criteria for Adverse Events (NCI CTCAE) v4.03 and reported by system organ class and preferred term using MedDRA dictionary version 22.0.

An erythroid response (pretreatment values less than 11 g/dL) was defined as hemoglobin increase by ≥ 1.5 g/dL, reduction of units of RBC transfusions by an absolute number of ≥ 4 RBC transfusions per 8 weeks, compared with the pretreatment transfusion number in the previous 8 weeks. Platelet response (pretreatment values < 100 × 10^9^/L) was defined as an absolute increase of ≥ 30 × 10^9^/L for patients starting with > 20 × 10^9^/L platelets; increase from < 20 × 10^9^/L to > 20 × 10^9^/L and by at least 100%. A neutrophil response (pretreatment values < 1.0 × 10^9^/L) was defined as ≥ 100% increase and an absolute increase of > 0.5 × 10^9^/L.

Continuous variables (i.e., patient characteristics, efficacy, and safety data) are summarized by descriptive statistics. Data summaries were produced using SAS version 9.4 (SAS Institute, Cary, NC, USA).

## Results

As of August 9, 2019, 40 patients were enrolled and treated in part 1, and 27 patients were enrolled and treated in part 2 (Online Resource 1). Patient demographics and baseline disease characteristics are summarized in Table 1. For durvalumab, the median duration of exposure was 18.0 weeks (range 4.0─121.9) in part 1 and 12.0 weeks (range 4.0─47.7) in part 2. For tremelimumab, the median duration of exposure in part 2 was 12.0 weeks (range 4.0─17.1). For azacitidine, the median duration of exposure in part 2 was 8.6 weeks (range 4.7─25.1) (Online Resource 2).

**Table 1:** Patient demographics and baseline disease characteristics, as-treated population

Characteristic	Part 1: durvalumab monotherapy			Part 2: durvalumab combination therapy			
	Low/int-1	Int-2/high	Total	Durva	Durva	Durva	Total
	(*n* = 18)	(*n* = 22)	(*N* = 40)	+ treme 25 mg	+ treme 75 mg	+ treme 75 mg + aza	(*N* = 27)
				**(*n*** **= 3)**	**(*n*** **= 17)**	**(*n*** **= 7)**	
**Age**, **years**	74.0 (54–96)	72.0 (48–87)	73.0 (48–96)	75.0 (73–83)	74.0 (52–84)	74.0 (72–84)	74.0 (52–84)
**Sex**, ***n*(%)**							
Male	11 (61)	18 (82)	29 (73)	3 (100)	12 (71)	6 (86)	21 (78)
**ECOG performance status**, ***n*(%)**							
0	6 (33)	8 (36)	14 (35)	0	4 (24)	0	1 (15)
1─2	12 (67)	14 (64)	26 (65)	3 (100)	13 (76)	7 (100)	23 (85)
**Median (range) bone marrow blasts**,** %**	4.0 (0–9)	9.5 (2–19)	6.0 (0–19)	8.5 (5–12)	9.0 (1–15)	5.5 (0–10.3)	7.0 (0–15)
**Median (range) platelets**,** 10**^**3**^**/µL**	148 (46–406)	59 (18–325)	100.5 (18–406)	11.0 (7–299)	33.0 (14–96)	54.0 (17–139)	33.0 (7–299)
**Median (range) ANC**,** 10**^**3**^**/µL**	1.5 (0.68–5.47)	1.33 (0.20–23.82)	1.44 (0.20–23.82)	0.44 (0–4.01)	0.65 (0.09–3.21)	0.96 (0.31–3.17)	0.80 (0–4.01)
**Hemoglobin**,** g/dL**	8.55 (6.4–11.7)	8.60 (7.1–11.9)	8.60 (6.4–11.9)	8.7 (8.6–10.3)	8.2 (6.6–11.6)	8.2 (7.1–9.3)	8.3 (6.6–11.6)
**Derived IPSS-R risk category**, ***n*(%)**							
Low (> 1.5─3)	6 (33.3)	1 (4.5)	7 (17.5)	0	0	0	0
Intermediate (> 3─4.5)	9 (50.0)	7 (31.8)	16 (40.0)	2 (66.7)	4 (23.5)	2 (28.6)	8 (29.6)
High (> 4.5─6)	3 (16.7)	8 (36.4)	11 (27.5)	0	6 (35.3)	2 (28.6)	8 (29.6)
Very High (> 6)	0	6 (27.3)	6 (15.0)	1 (33.3)	7 (41.2)	3 (42.9)	11 (40.7)
**Karyotype classification (adjudicated)**, ***n*(%)**							
Normal	10 (55.6)	6 (27.3)	16 (40.0)	1 (33.3)	4 (23.5)	1 (14.3)	6 (22.2)
Single	6 (33.3)	5 (22.7)	11 (27.5)	1 (33.3)	8 (47.1)	3 (42.9)	12 (44.4)
Loss or del 5	2 (11.1)	0	2 (5.0)	0	2 (11.8)	1 (14.3)	3 (11.1)
Trisomy 8	1 ( 5.6)	1 (4.5)	2 (5.0)	1 (33.3)	0	0	1 (3.7)
Other	3 (16.7)	4 (18.1)	7 (17.5)	0	6 (35.3)	2 (28.6)	8 (29.6)
Double	2 (11.1)	5 (22.7)	7 (17.5)	0	2 (11.8)	1 (14.3)	3 (11.1)
Complex	0	6 (27.3)	6 (15.0)	1 (33.3)	2 (11.8)	2 (28.6)	5 (18.5)
Unknown	0	0	0	0	1 (5.9)	0	1 ( 3.7)
**Time from diagnosis**,** months (range)**	20.4 (0.2–185.2)	19.6 (0.6–93.8)	20.4 (0.2–185.2)	13.4 (12.6–18.6)	20.8 (7.0–115.7)	17.2 (5.9–97.3)	17.2 (5.9–115.7)
**Number (range) of prior treatments**	1.0 (1–4)	1.0 (1–4)	1.0 (1–4)	1.0 (1–1)	1.0 (1–3)	1.0 (1–3)	1.0 (1–3)
**Prior treatments**, ***n*(%)**							
Chemotherapy	17 (94)	22 (100)	39 (98)	3 (100)	17 (100)	7 (100)	27 (100)
Azacitidine	12 (67)	18 (82)	30 (75)	2 (67)	16 (94)	5 (71)	23 (85)
Decitabine	11 (61)	6 (27)	17 (43)	1 (33)	2 (12)	3 (43)	6 (22)
Lenalidomide	2 (11)	3 (14)	5 (13)	0	1 (6)	1 (14)	2 (7)
Other	1 (6)	2 (9)	3 (8)	0	2 (12)	1 (14)	3 (11)
Biologic	3 (17)	0	3 (8)	0	0	0	0
Other	0	4 (18)	4 (10)	0	1 (6)	2 (29)	3 (11)

*ANC* absolute neutrophil count, *aza* azacitidine, *durva* durvalumab, *ECOG* Eastern Cooperative Oncology Group, *int* intermediate, *IPSS* International Prognostic Scoring System, *treme* tremelimumab. Aranesp and epogen were considered ‘Biologics’, whereas thalidomide, R-CHOP, and vorinostat were considered as ‘Other’

The recommended dose of durvalumab (10 mg/kg Q2W) was chosen based on the data from the part 1 dose-escalation phase. No DLTs were observed in part 1. In part 2, there were 3 (11%) patients who experienced DLTs (Grade 3 rash maculo-papular [arm 1/cohort 2]; Grade 3 enterocolitis [arm 2]; Grade 3 myocarditis [arm 2]). The most common treatment-emergent AEs (TEAEs) were fatigue (44%), anemia (37%), and pyrexia (30%) (Online Resource 3). Treatment-related AEs were reported in 18 (66.7%) patients. The most common treatment-related AEs were fatigue (22.2%) and diarrhea (18.5%). A total of 7 (26%) deaths were due to serious AEs. One death was considered related to both durvalumab and tremelimumab by the investigator (myocarditis, *n* = 1).

### Part 1

In part 1, 35% (14/40) of patients had bone marrow blasts < 5% at baseline. No patients had confirmed complete response (CR) or confirmed partial response (PR) following treatment with durvalumab monotherapy. 15% (6/40) of patients had a best overall response (BOR) of marrow complete response (mCR), and 35% (14/40) of patients had a BOR of stable disease (Table 2). Of the 6 patients with mCR, one had HI-neutrophil (HI-N). Progressive disease (PD) was observed in 8% (3/40) of patients, all of whom were in the intermediate-2/high-risk group and experienced PD at least 3 months after treatment (days 99, 101, and 105); no patients had their disease transform to AML. Blast reductions were observed in 3 patients in the low/intermediate-1 risk group: from 5─10% to 2%, 6% to 2%, and 8% to 3%. In the intermediate-2/high-risk group, 3 patients achieved blast reductions: from 6% to 1%, 10% to4%, and 11% to 3%. The disease control rate (mCR + stable disease; no CRs or PRs observed) was 61% (11/18) in the low/intermediate-1 risk group and 41% (9/22) in the intermediate-2/high-risk group. In total, HI was observed in 35% (14/40) of patients in part 1. Following HI, 21% (3/14) of patients experienced PD/relapse (Table 2). The duration of follow-up for these 3 patients was 27.1, 47.9, and 51.4 weeks, respectively.

**Table 2:** Disease response, as-treated population

Parameter	Part 1: durvalumab monotherapy			Part 2: durvalumab combination therapy			
	Low/int-1	Int-2/high	Total	Durva	Durva	Durva	Total
	(*n* = 18)	(*n* = 22)	(*N* = 40)	+ treme 25 mg	+ treme 75 mg	+ treme 75 mg	(*N* = 27)
				**(*n*** **= 3)**	**(*n*** **= 17)**	+ aza	
						**(*n*** **= 7)**	
**Best overall response**,^a^*n*(%)							
CR	0	0	0	0	0	0	0
PR	0	0	0	0	0	0	0
mCR	3 (17)	3 (14)	6 (15)	1 (33)	2 (12)	1 (14)	4 (15)
SD	8 (44)	6 (27)	14 (35)	0	1 (6)	1 (14)	2 (7)
PD	0	3 (14)	3 (8)	0	2 (12)	0	2 (7)
Transformation to AML	0	0	0	0	1 (6)	0	1 (4)
Non-PR/Non-PD	5 (28)	9 (41)	14 (35)	1 (33)	8 (47)	1 (14)	10 (37)
Non-evaluable	2 (11)	1 (5)	3 (8)	1 (33)	4 (24)	4 (57)	9 (33)
**OR (confirmed CR + mCR + PR),** *n*(%)	3 (17)	3 (14)	6 (15)	1 (33)	2 (12)	1 (14)	4 (15)
95% CI	(3.6, 41.4)	(2.9, 34.9)	(5.7, 29.8)	(0.8, 90.6)	(1.5, 36.4)	(0.4, 57.9)	(4.2, 33.7)
**HI, ***n*/*m*(%)^b^	5/18 (28)	9/22 (41)	14/40 (35)	0/3 (0)	7/17 (41)	1/7 (14)	8/27 (30)
HI-E	3/18 (17)	2/22 (9)	5/40 (13)	0/3 (0)	1/17 (6)	0/7 (0)	1/27 (4)
HI-P	1/10 (10)	3/18 (17)	4/28 (14)	0/2 (0)	4/17 (24)	0/7 (0)	4/26 (15)
HI-N	3/11 (27)	4/19 (21)	7/30 (23)	0/2 (0)	5/15 (33)	1/6 (17)	6/23 (26)
Progression/relapse after HI, *n*/*m*(%)^c^	2/5 (40)	1/9 (11)	3/14 (21)	0/0	0/7 (0)	0/1 (0)	0/8 (0)
HI-E	1/3 (33)	0/2 (0)	1/5 (20)	0/0	0/1 (0)	0/0	0/1 (0)
HI-P	0/1 (0)	0/3 (0)	0/4 (0)	0/0	0/4 (0)	0/0	0/4 (0)
HI-N	1/3 (33)	1/4 (25)	2/7 (29)	0/0	0/5 (0)	0/1 (0)	0/6 (0)
PFS,^d^months, median (80% CI)	22.3 (11.9, 24.3)	6.0 (4.0, 7.4)	9.0 (7.4, 11.4)	9.3 (2.2, 11.5)	3.7 (3.6, 4.1)	NR (NE, NE)	3.8 (3.7, 6.6)
6-month PFS rate,^e^(80% CI)	88.5 (74.0, 95.2)	50.0 (35.8, 62.7)	66.5 (55.8, 75.2)	66.7 (22.9, 89.4)	25.5 (12.1, 41.2)	60.0 (27.9, 81.5)	38.1 (24.9, 51.2)
OS,^f^months, median (80% CI)	23.8 (15.5, 27.7)	8.0 (5.5, 9.3)	11.8 (9.2, 14.1)	9.3 (2.2, 11.5)	5.3 (3.7, 6.8)	NR (NE, NE)	5.3 (3.7, 7.6)
6-month OS rate,^e^(80% CI)	88.9 (74.7, 95.4)	59.1 (44.5, 71.1)	71.7 (61.3, 79.8)	66.7 (22.9, 89.4)	43.2 (27.0, 58.4)	60.0 (27.9, 81.5)	49.9 (36.0–62.2)

*AML* acute myeloid leukemia, *aza* azacitidine, *CI* confidence interval, *CR* complete response, *durva* durvalumab; *HI* hematologic improvement, *HI-E* HI-erythroid, *HI-N* HI-neutrophil, *HI-P* HI-platelet, *int* intermediate, *IWG* International Working Group, *mCR* marrow complete response, *MDS* myelodysplastic syndrome, *NR* median estimate could not be calculated, *NE* not estimable, *OS* overall survival, *PD* progressive disease, *PFS* progression-free survival, *PR* partial response, *SD* stable disease

^a^Response as defined by the IWG 2006 MDS response criteria

^b^The denominator “m” for each type of HI is the number of patients who were abnormal at baseline for each cell lineage (E, P, N)

^c^The denominator “m” for each type of relapse/progression HI is the number of patients who had that type of HI

^d^Not censored for subsequent anticancer therapy. Median PFS was assessed using the Kaplan-Meier method

^e^Rates were estimated using the Kaplan-Meier method, and their confidence intervals were calculated using the log-log transformation

^f^Median OS was assessed using the Kaplan-Meier method

**Fig. 1:**
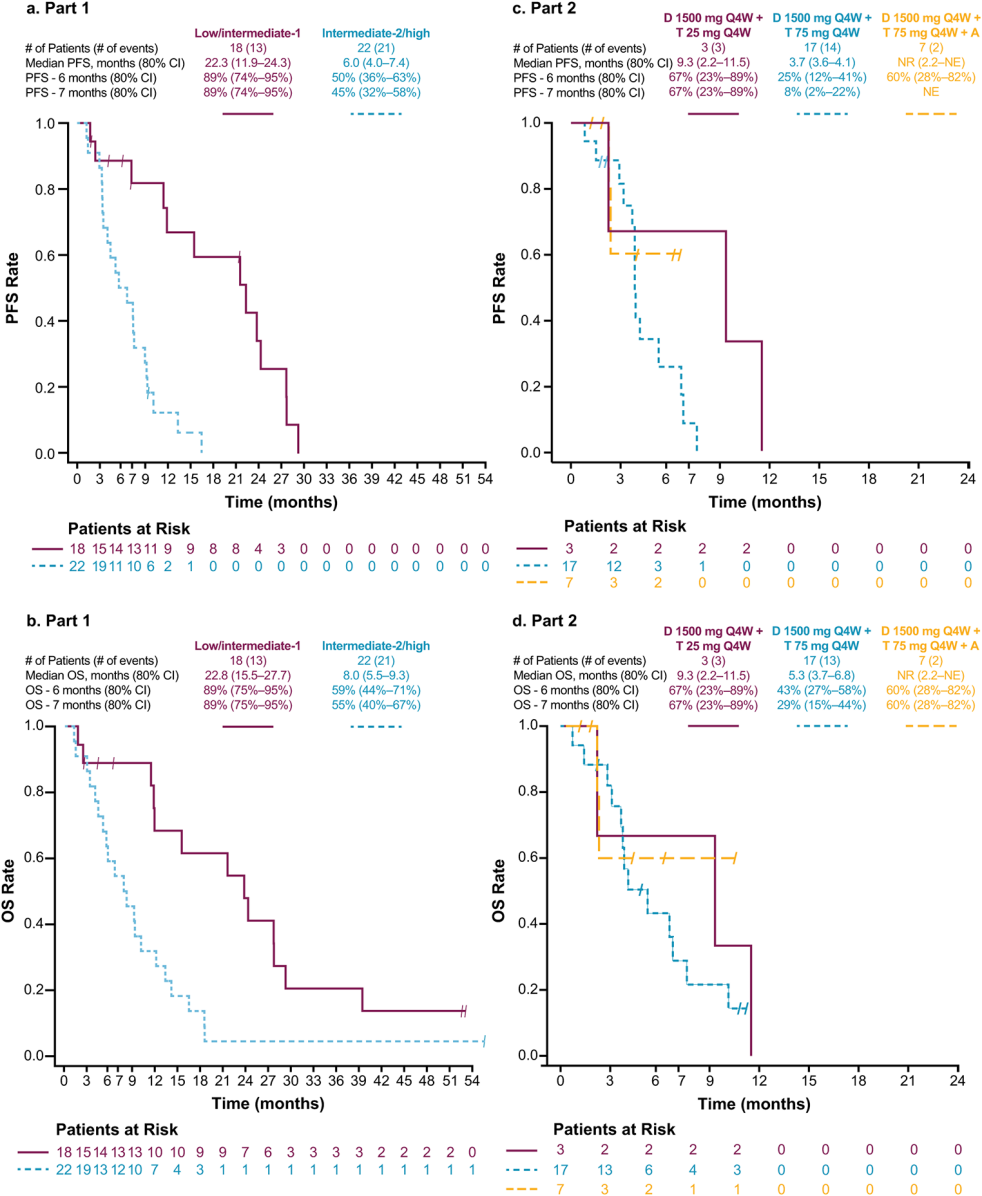
Survival, as-treated population. **A** Progression-free survival, part 1. **B** Overall survival, part (1) **C**. Progression-free survival, part (2) **D** Overall survival, part 2. *A* azacitidine, *D* durvalumab, *CI* confidence interval, *NE* not estimable, *NR* median estimate could not be calculated, *PFS* progression-free survival, *OS* overall survival, *Q4W* every 4 weeks, *T* tremelimumab

Median progression-free survival (PFS) for patients in Part 1 was 22.3 months (80% CI, 11.9─24.3) for the low/intermediate-1 risk group and 6.0 months (80% CI, 4.0─7.4) for the intermediate-2/high-risk group (Fig. 1A). Median overall survival (OS) was 23.8 months (80% CI, 15.5─27.7) for the low/intermediate-1 risk group and 8.0 months (80% CI, 5.5─9.3) for the intermediate-2/high-risk group (Fig. 1B).

### Part 2

In part 2 of the study, 25.9% (7/27) of patients had bone marrow blasts < 5% at baseline. No patients had confirmed CR or confirmed PR following treatment with durvalumab in combination with tremelimumab, with or without azacitidine. Stable disease was reported in 7% (2/27) of patients and mCR was reported in 15% (4/27) of patients (Table 2). Of the 4 patients who had mCR, 1 patient had HI-N. PD was observed in 7% (2/27) of patients, and 4% (1/27) of patients had disease transformation to AML. A blast reduction from 20% to 4% was observed in 1 patient in the durvalumab/tremelimumab 25-mg cohort. In the durvalumab/tremelimumab 75-mg cohort, 2 patients achieved blast reductions from 15─19% to 3% and from 11% to 2%. In the durvalumab/tremelimumab 75-mg/azacitidine cohort, 1 patient achieved a blast reduction from 10─125 to 0%.

The disease control rate (mCR + stable disease; no CRs or PRs observed) was 33% (1/3) in the durvalumab/tremelimumab 25-mg cohort; 18% (3/17) in the durvalumab/tremelimumab 75-mg cohort; and 29% (2/7) in the durvalumab/tremelimumab 75-mg/azacitidine cohort. HI was observed in 30% (8/27) of patients. Following HI, no patients experienced PD/relapse as defined by IWG 2006 MDS criteria.

Median PFS was: 9.3 months (80% CI, 2.2─11.5) in the durvalumab/tremelimumab 25-mg cohort; 3.7 months (80% CI, 3.6─4.1) in the durvalumab/tremelimumab 75-mg cohort; and not reached (NR) (80% CI, 2.2─not estimable [NE]) in the durvalumab/tremelimumab 75-mg/azacitidine cohort (Fig. 1C). Median OS was 9.3 months (80% CI, 2.2─11.5) in the durvalumab/tremelimumab 25-mg cohort; 5.3 months (80% CI, 3.7─6.8) in the durvalumab/tremelimumab 75-mg cohort; and NR (80% CI, 2.2─NE) in the durvalumab/tremelimumab 75-mg/azacitidine cohort (Fig. 1D).

## Discussion

In this phase 1 study, 3 DLTs were observed (all in part 2), and there were no unexpected toxicities or new safety concerns following durvalumab treatment (as monotherapy, with or without azacitidine, or part of combination therapy with tremelimumab, with or without azacitidine) in patients with MDS who had previously been treated with HMAs. The incidence of treatment-related AEs was similar to what was previously observed in patients with solid tumors treated with durvalumab [23] Durvalumab PK parameters following monotherapy and in combination with tremelimumab, with or without azacitidine, were consistent with the PK profile for durvalumab in patients receiving the same durvalumab dosing regimen for other tumor types. Clinical activity of durvalumab monotherapy was limited to maintenance of stable disease; however, HI was observed in 35% of patients who received durvalumab monotherapy. This study was terminated early due to limited efficacy. No patients had confirmed CR or confirmed PR. This is consistent with the final results of a phase 2 clinical trial of azacitidine with and without durvalumab in patients with high-risk MDS, in which no meaningful difference in efficacy was observed between treatments for either cohort [15]. Notably, 15% of patients had mCRs in this study (part 1, 6/40; part 2, 4/27). The results of the mutational analysis for patients in part 1 of the study revealed an increased frequency of mutations typically associated with MDS diagnosis, such as *ASXL1*, *SRSF2*, and *TET2.* [24] However, data were not sufficient to make conclusions on the relationship between the presence of mutations and clinical response.

The limited clinical activity observed in this study may be attributed to several factors. In MDS, the tumor microenvironment differs from that of solid tumors, with high numbers of myeloid-derived suppressor cells [25]. Suppressive mechanisms of natural killer cells, T cells, FOXP3 + regulatory T cells, and myeloid-derived suppressor cells may impair T-cell function in patients with MDS [26] While immunosuppression (via T-cell depletion, antithymocyte globulin, and alemtuzumab) is an effective therapy for some patients with MDS, the exact mechanism remains unclear, and selection of patients who may benefit remains a challenge [7]. Lastly, blasts typically have a lower tumor mutational burden compared with solid tumors, and low tumor mutational burden has been associated with reduced efficacy of immune checkpoint therapy [27].

Some limitations of the study included outcomes not being stratified by baseline PD-1/PDL-1 expression, and the lack of correlative PD-1 analyses. Additional studies are warranted to better understand the full potential of immune checkpoint therapies in the treatment of MDS, as single agents and in combination with other types of therapy, and to better identify subsets of patients who could potentially derive a clinical benefit from these types of therapies.

## Electronic supplementary material

Below is the link to the electronic supplementary material.

Supplementary Material 1

## Data Availability

Data underlying the findings described in this manuscript may be obtained in accordance with AstraZeneca’s data sharing policy described at: https://www.astrazenecagrouptrials.pharmacm.com/ST/Submission/Disclosure. Data for studies directly listed on Vivli can be requested through Vivli at https://www.vivli.org. Data for studies not listed on Vivli could be requested through Vivli at https://www.vivli.org/members/enquiries-about-studies-not-listed-on-the-vivli-platform. AstraZeneca Vivli member page is also available outlining further details: https://www.vivli.org/ourmember/astrazeneca/.

## Declarations

### Clinical trial registration

This study is registered with ClinicalTrials.gov, NCT02117219.
