# Supplementary material for: A phase 1 study of durvalumab as monotherapy or combined with tremelimumab with or without azacitidine in patients with myelodysplastic syndrome
Source: Ann Hematol. 2025 Mar 28;104(3):1577–85. doi: 10.1007/s00277-024-06081-4 (PMC12031784; doi:10.1007/s00277-024-06081-4)
Supplement: Supplementary file 1 — Supplementary Material 1 [file 277_2024_6081_MOESM1_ESM.pdf]

Supplementary Figures and Tables

Online Resource 1.

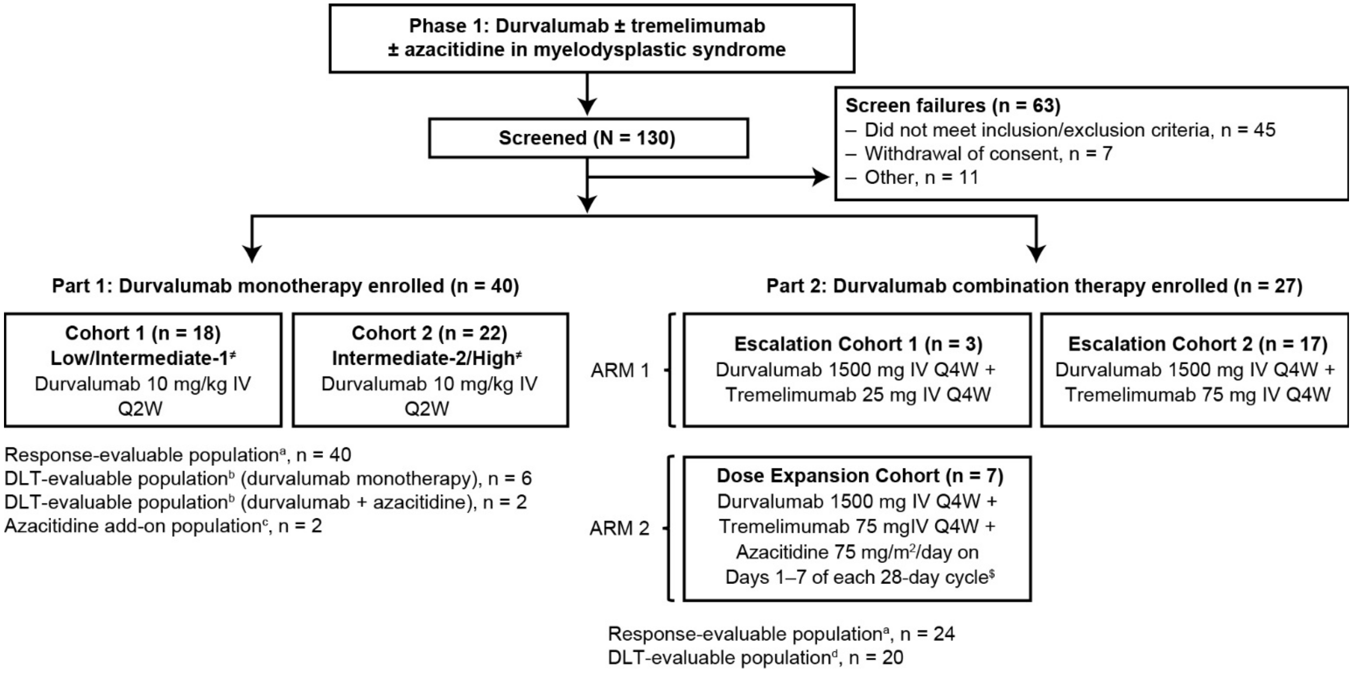

Online Resource 1. Study design. [ǂ]IPSS risk status; * SC injection. [a]Response-evaluable population includes patients in the as-treated population who had ≥1 post-baseline bone marrow biopsy, who died from any cause, or who discontinued due to clinical PD prior to any post-baseline bone marrow biopsy. [b]The initial 6 patients enrolled in the study who received ≥2 protocol-assigned doses of durvalumab and completed the safety follow-up through the DLT evaluation period or experienced a DLT during the DLT evaluation period. [c]Azacitidine add-on population includes patients in part 1 who received durvalumab and azacitidine (75 mg/m^2/day on days 1 to 7 of each 28-day cycle) after initial treatment with durvalumab monotherapy. [d]DLT-evaluable population includes all patients entered in the dose-escalation phase who received ≥2 doses of durvalumab and completed the safety follow-up through the DLT evaluation period (through the administration of the third dose of durvalumab and tremelimumab with or without azacitidine) or experienced any DLT.

DLT dose-limiting toxicity, *durva* durvalumab, *IPSS* International Prognostic Scoring System, *IV* intravenous, *PD* progressive disease, *Q2W* every 2 weeks, *Q4W* every 4 weeks, *SC* subcutaneous.

Online Resource 2. Duration of exposure, as-treated population.

	**Part 1: durvalumab monotherapy**			**Part 2: durvalumab combination therapy**			
	**Low/int-1 (*n* = 18)**	**Int-2/high (*n* = 22)**	**Total (*N* = 40)**	**Durva + treme 25 mg (*n* = 3)**	**Durva + treme 75 mg (*n* = 17)**	**Durva + treme 75 mg + aza (*n* = 7)**	**Total (*N* = 27)**
**Duration of exposure,[a] weeks, median (range)**							
**Durvalumab**	26.2 (6.0, 121.9)	13.0 (4.0, 56.0)	18.0 (4.0, 121.9)	12.3 (12.0, 47.7)	13.0 (4.0, 32.0)	8.0 (4.0, 24.3)	12.0 (4.0, 47.7)
**Tremelimumab**	-	-	-	12.0 (12.0, 12.3)	13.0 (4.0, 17.1)	8.0 (4.0, 16.0)	12.0 (4.0, 17.1)
**Azacitidine**	19.7 (19.7, 19.7)	9.0 (9.0, 9.0)	14. 4 (9.0, 19.7)	-	-	8.6 (4.7, 25.1)	8.6 (4.7, 25.1)

[a]Duration of exposure is defined by the last dose date, plus 28 days, minus the first dose date. For patients who die prior to day 28 of the last cycle, duration is defined as the date of death, minus first dose date, plus 1 day. If the database cutoff occurs prior to day 28 of the last cycle, and the subject remains alive, duration is defined as the date of the database cutoff, minus the dose date, plus 1 day.
Aza azacitidine, *durva* durvalumab, *int* intermediate, *treme* tremelimumab.

Online Resource 3. Treatment-emergent adverse events of any grade in ≥20% of all patients in either part 1 or part 2, as-treated population.

**System Organ Class[a]**<br>**Preferred term**	**Part 1: durvalumab monotherapy**			**Part 2: durvalumab combination therapy**			
	**Low/int-1 (*n* = 18)**	**Int-2/high (*n* = 22)**	**Total (*N* = 40)**	**Durva + treme 25 mg (*n* = 3)**	**Durva + treme 75 mg (*n* = 17)**	**Durva + treme 75 mg + aza (*n* = 7)**	**Total (*N* = 27)**
**Any treatment-emergent AE**	18 (100)	22 (100)	40 (100)	3 (100)	17 (100)	7 (100)	27 (100)
**Blood and lymphatic system disorders**							
Anemia	4 (22)	8 (36)	12 (30)	1 (33)	7 (41)	2 (29)	10 (37)
Febrile neutropenia	-	-	-	1 (33)	4 (24)	2 (29)	7 (26)
Thrombocytopenia	2 (11)	6 (27)	8 (20)	-	-	-	-
**Gastrointestinal disorders**							

Abdominal pain	1 (6)	4 (18)	5 (13)	0	5 (29)	1 (14)	6 (22)
Diarrhea	7 (39)	9 (41)	16 (40)	0	4 (24)	2 (29)	6 (22)
Nausea	3 (17)	5 (23)	8 (20)	0	5 (29)	0	5 (19)
**General disorders and administration site conditions**							
Chills	0	6 (27)	6 (15)	2 (67)	2 (12)	2 (29)	6 (22)
Edema peripheral	4 (22)	7 (32)	11 (28)	0	4 (24)	3 (43)	7 (26)
Fatigue	6 (33)	10 (46)	16 (40)	1 (33)	7 (41)	4 (57)	12 (44)
Pyrexia	4 (22)	11 (50)	15 (38)	0	7 (41)	1 (14)	8 (30)
**Metabolism and nutrition disorders**							
Hyperglycemia	5 (28)	3 (14)	8 (20)	-	-	-	-
**Respiratory, thoracic, and mediastinal disorders**							
Cough	3 (17)	9 (41)	12 (30)	1 (33)	5 (29)	1 (14)	7 (26)

Dyspnea	1 (6)	9 (41)	10 (25)	0	5 (29)	1 (14)	6 (22)
**Skin and subcutaneous tissue disorders**							
Rash maculo-papular	2 (11)	2 (9)	4 (10)	2 (67)	3 (18)	1 (14)	6 (22)

AE adverse event, *Aza* azacitidine, *durva* durvalumab, *int* intermediate, *treme* tremelimumab.

[a]Patients were counted once for each System Organ Class and preferred term, regardless of the number of events.

Data are presented as n (%).

MedDRA version 22.0.
